# Visibility of Recurrent Caries Through Universal Shade Resin Composite Restorations

**DOI:** 10.3390/ma17235815

**Published:** 2024-11-27

**Authors:** Ryotaro Yago, Chiharu Kawamoto, Di Wu, Takuma Mirokuin, Rafiqul Islam, Monica Yamauti, Hidehiko Sano, Atsushi Tomokiyo

**Affiliations:** 1 Department of Restorative Dentistry, Graduate School of Dental Medicine, Hokkaido University, Kita 13 Nishi 7, Kita-ku, Sapporo 060-8586, Japan; adjmtw9165@gmail.com; 2 Department of Restorative Dentistry, Faculty of Dental Medicine, Hokkaido University, Kita 13 Nishi 7, Kita-ku, Sapporo 060-8586, Japan; wudi0526@den.hokudai.ac.jp (D.W.);; 3 Department of Biomedical and Applied Science, School of Dentistry, Indiana University, 1121 W. Michigan St., Indianapolis, IN 46202, USA; myamauti@gmail.com

**Keywords:** recurrent caries, caries detection, resin composite, universal shade resin composite, structural color

## Abstract

This in vitro study aimed to investigate whether color differences in the stained cavity floor simulating recurrent caries can be detected using various restored resin composites. Artificial teeth were made with conventional resin composite (Estellite Sigma Quick A3; ET), and class V cavities were prepared. To simulate the color of caries, a dot was marked in the center of the cavity floor using four different magic pens. The cavities were filled with ET and two universal shade resin composites (UC, Omnichroma; OMI, Essentia Universal; ESS). For photographic analysis, a standard correction color chart was used. The color difference (Δ*E*_00_) between (i) the lab value at the center of the cavity and (ii) the average lab value at 1 mm mesial and distal to the center of the cavity was calculated. The data were statistically746o-way ANOVA (*p* < 0.05). For visual analysis, 25 dentists were asked to complete a questionnaire to determine whether the color differences were noticeable. In the photographic analysis, UC showed larger Δ*E*_00_ values than ET in all colors. Visual analysis revealed higher detection rates for UC than ET. Universal shade resin composite tends to reflect the color of the cavity more effectively than conventional resin composite.

## 1. Introduction

In recent years, resin composites have been used extensively because of their improved mechanical strength and bond strength, as well as their excellent esthetic qualities [1,2,3]. However, in some clinical situations, recurrent caries (secondary caries) may occur but not break away, delaying the detection of caries in some cases.

According to a Japanese government report, more than 90% of adults in Japan have experienced dental caries in the past, 30% of adults have untreated dental caries, and dental caries is the cause of approximately 40% of tooth extractions in people over 40 years old [4]. One of the characteristics of adult dental caries is recurrent caries [5]. Recurrent caries is more likely to progress deeper into the dentin and is therefore more likely to lead to pulpitis [6]. The government cautions that pulp-treated teeth are less likely to be painless, further delaying the detection of caries [6].

Recurrent caries is usually detected through visual and radiographic examinations [7,8,9,10]. Early detection of recurrent caries allows minimal intervention and the conservation of more tooth structures [11]. In the case of recurrent caries under a resin composite, if patients can look at their teeth in a mirror and notice changes, it is expected to trigger a visit to the dentist, leading to early detection and treatment. It may also lead to an improvement in the sensitivity of dentists’ visual examination.

Dental caries can be classified into acute and chronic caries [12]. Acute caries is more common in young people, while chronic caries is more common in middle-aged and older adults [13]. The color of softened dentin in acute caries is pale yellow, whereas that in chronic caries is brown to blackish brown [14].

In recent years, universal shade resin composite (UC) has been developed to match the color of teeth with a single paste [15,16]. They are based on structural color [17] or light-scattering properties [18]. Conventional resin composites (non-UC) achieve a good color match with the tooth structure by adding pigments [17,19,20].

Omnichroma (OMI) is a UC based on structural color that develops by selectively interacting with the nanostructure of the tooth [17]. The uniform spacing and arrangement of spherical OMI particles facilitates the transmission of light throughout the restoration [21]. It reflects the color of the cavity surrounding the restoration and obtains a color match through blending [22].

Essentia Universal (ESS) is a microhybrid UC with a blending effect [23]. The light transmission and scattering properties of ESS with microhybrid fillers are known to blend naturally with the surrounding tooth structure [24].

Estellite Sigma Quick (ET) is a non-UC and contains pigment.

When targeting the color of caries, the use of natural teeth would make evaluation between materials difficult due to the large variation in color. Therefore, we used a method to determine the detection ability of caries color under uniform color conditions using magic pens and resin composites.

The purpose of this study was to simulate artificial caries in a standardized class 5 cavity using a magic pen and filling it with UC and non-UC and (1) to measure the color difference after filling (2) to conduct a questionnaire survey to dentists to determine if the color difference was obvious by visual examination.

The null hypotheses are (1) there is no color difference due to the difference in the color given, (2) there is no color difference due to the difference in the material, and (3) the difference in the material affects the detection rate due to the difference in the operator.

## 2. Materials and Methods

### 2.1. Preparation of Specimens

**Figure 1:**
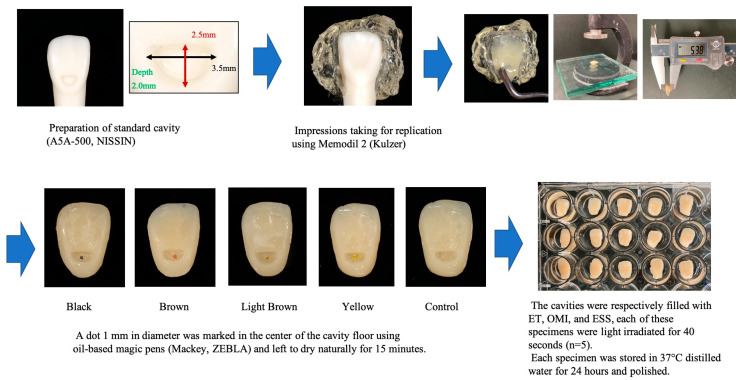
Sample preparation procedures of the specimens.

The materials used in the study are listed in Table 1. In this study, ET (A3 shade, Tokuyama Dental, Tokyo, Japan) was used as the conventional resin composite, and OMI (Tokuyama Dental) and ESS (GC, Tokyo, Japan) were used as the UCs. The specimen preparation procedure is shown in Figure 1. A standard cavity of 2.5 mm in length, 3.5 mm in width, and 2.0 mm in depth in an artificial tooth (A5A-500, Nissin, Kyoto, Japan) was prepared using a diamond (FG 401, Shofu, Kyoto, Japan). A replica impression was made using silicon material (Memodil 2, Kulzer, Hanau, Germany). The surface of the impression was filled with ET and then irradiated for 40 s using a light irradiator (Pencure 2000, Morita, Tokyo, Japan; irradiation intensity 2000 mW/cm^2^).

**Figure 2:**
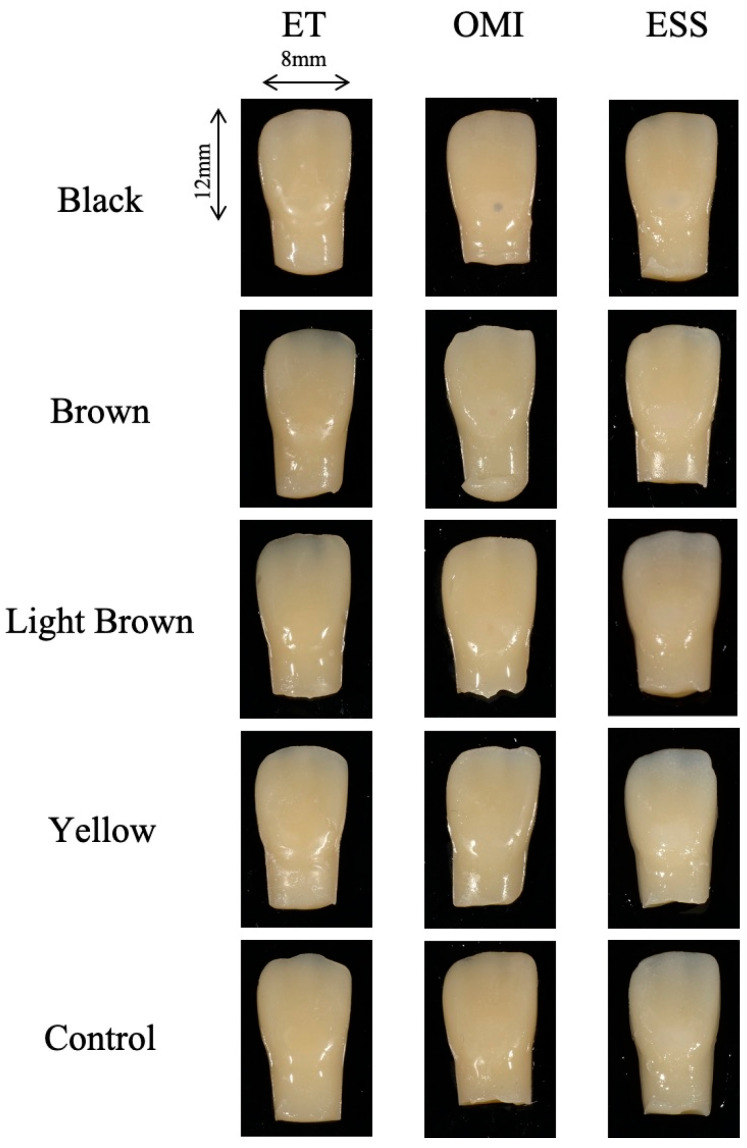
Specimens of each group after polishing.

To simulate caries color, a dot (approximately 1 mm) in diameter was marked at the center of the cavity floor with four color (black, brown, light brown, and yellow) oil-based magic pens (Mackey, Zebla, Tokyo, Japan) and left to dry for 15 min. To standardize the location of the dots, the center of the cavity was determined with a stereomicroscope when coloration was applied. The cavities were incrementally filled with ET, OMI, or ESS without adhesive application and irradiated with light for 40 s. For the control group, resin teeth without markings were prepared. Each sample was stored in distilled water at 37 °C for 24 h. The specimens were polished with a silicon point (White Point CA, Shofu) and a soft polisher (Super Snap, Shofu) for 30 s each and stored in water. The specimens from each group (*n* = 5) are shown in Figure 2.

### 2.2. Photographic Analysis

**Figure 3:**
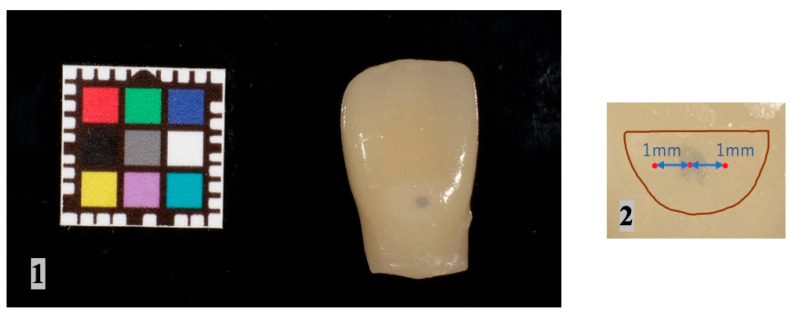
Photographic analysis. **1**: Photographed using Casmatch. **2**: CIE L*a*b* was recorded at the center of the cavity and 1 mm mesial and distal to the center of the cavity.

Photographic analysis was performed using image-corrected color cards (CASMATCH, Bearmedic, Ibaraki, Japan). Photographic conditions included a darkroom, a shooting distance of 100 mm, and a black background. Representative images are shown in Figure 3 panel (1). Image editing software (Adobe Photoshop 2021, Adobe, San Jose, CA, USA) was used for image analysis. To calibrate the color of the image, the sampling range of the eyedropper tool was adjusted to the average of a 5 × 5 pixels square. Correcting contrast and gray balance by setting reference values for color level correction produces images with consistent tone levels. CIE L*a*b* and CIEDE 2000 color difference formulas were used for color evaluation [25,26]. CIE L*a*b* values were recorded at the center of the cavity and at points 1 mm mesial and distal to the center of the cavity (Figure 3 panel (2)). To eliminate bias due to measurement points, each sample was measured three times and the average value was used as the data. The color difference (Δ*E*_00_) between (i) the lab value at the center of the cavity and (ii) the average lab value at 1 mm mesial and distal to the center of the cavity was calculated using the CIEDE 2000 Formula (1).

(1) $$\Delta E_{00}={\left[\left(\frac{\Delta L^{\prime }}{kLSL}\right)2+\left(\frac{\Delta C^{\prime }}{kCSC}\right)2+\left(\frac{\Delta H^{\prime }}{kHSH}\right)2+RT\left(\frac{\Delta C^{\prime }}{kLSC}\right)\left(\frac{\Delta H^{\prime }}{kHSH}\right)\right]}^{1/2}$$

### 2.3. Visual Analysis

For visual analyses, one sample was randomly selected from each group to form three irregularly arranged plates. A total of 25 dentists and 25 volunteers were included in the study. The 25 licensed dentists were not less than 24 years of age, had less than 40 years of post-graduation experience, had bilateral naked eye visual acuity ≥ 0.6 or corrected visual acuity ≥ 0.8, and were free of color blindness or strong color deficiencies. The details are shown in Table A1, Table A2 and Table A3 (Appendix A). The 25 volunteers had to be at least 20 years old, with bilateral naked eye visual acuity ≥ 0.6 or corrected visual acuity ≥ 0.8, and without color blindness or strong color weakness, and not be a licensed dentist, dental hygienist, or dental technician. The details are shown in Table A4 and Table A5. The test was conducted in a double-blind manner. Each sample was visually inspected for significant color differences. First, the subjects were shown an unfilled control sample. They were then shown the plates and asked to judge within 2 s for each sample whether “the color difference is noticeable” or “do not know, cannot judge”, and to fill out the questionnaire. Finally, they were asked to vote for the sample with the most noticeable color difference in each plate.

### 2.4. Statistical Analysis

Two-way ANOVA was used to examine the effects of three different materials and five different colors (including the control) on Δ*E*_00_. When the interaction was significant, Bonferroni’s method was used to examine the simple main effect at each factor level. The Bonferroni method was used for post-hoc tests. SPSS ver. 29.0 (IBM, Armonk, NY, USA) was used for statistical analysis, with a significance level of 5%.

## 3. Results

**Figure 4:**
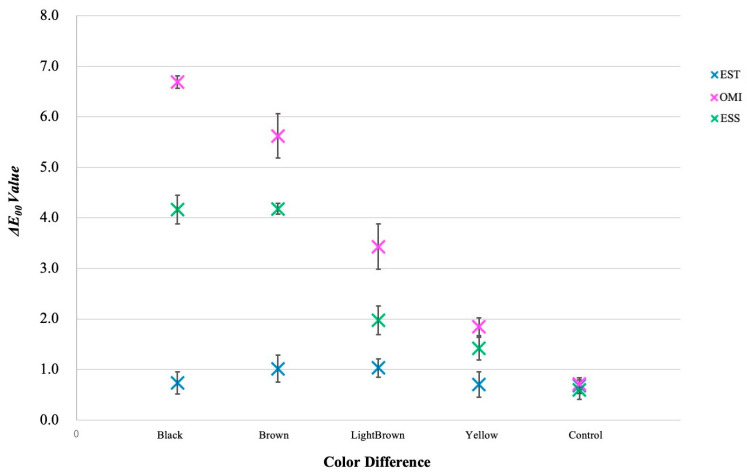
The mean and standard deviation of Δ*E*_00_ for each group of photographic analysis data. Two-way analysis of variance revealed a significant interaction between the three materials and the five colors (F (8, 74) = 122.19, *p* < 0.001). The Bonferroni method was used to examine the simple main effect for each level of each factor, and no significant differences were found between the five colors in the ET. In the OMI, there were significant differences among all five colors (*p* < 0.001), and in the ESS, there were significant differences among all groups, except between Brown and Black (*p* < 0.01). No significant differences were found between the ET, OMI, and ESS groups and the control group.

Figure 4 shows for every group of photographic data the mean and standard deviation of Δ*E_00_*. The three materials and the five colors (including the control) exhibited a substantial interaction in the two-way ANOVA [F (8, 74) = 122.19, *p* < 0.001]. The Bonferroni method investigates straightforward main effects at each factor level. The ET group did not exhibit any significant differences among the five colors, including the control. All categories in the ESS group exhibited a significant difference (*p* < 0.01), except between the Brown and Black groups. Among the ET, OMI, and ESS groups, the control group exhibited no significant differences.

**Figure 5:**
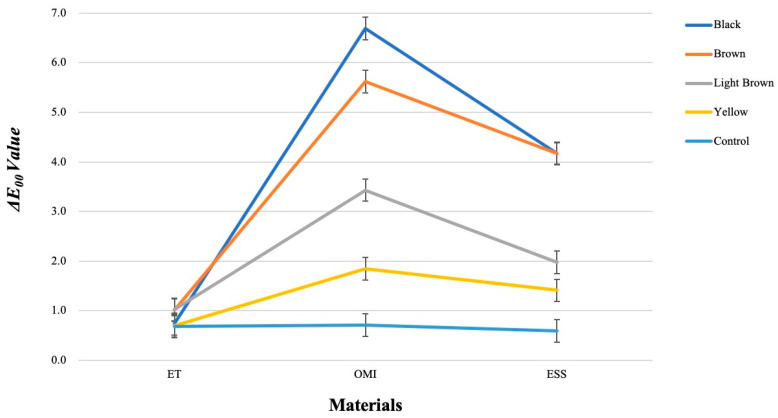
Estimated marginal means of Δ*E*_00_. Error bar: 95% CI. The trend of Δ*E*_00_ among the five colors of the materials is different, and there appears to be a difference between the colors in OMI and ESS, except between Black–Brown in ESS. However, it can be seen that there is less difference between the five colors in ET. This difference in the Δ*E*_00_ trends among the five colors of the three materials indicates an interaction. The graph also shows that Black, Brown, Light Brown, and Yellow have the same data trend of ET < ESS < OMI, while Control does not show such a clear trend. Thus, the difference in the trends of ET, ESS, and OMI among the five colors indicates the meaning of the interaction effect.

Figure 5 shows the estimated peripheral averages of Δ*E_00_*. The graph indicates that the trend of Δ*E_00_* among the five colors varied among materials. There appears to be a difference between the colors in OMI and ESS, except between the Black and Brown in ESS. However, there was less distinction between the five colors in the ET. The Δ*E_00_* difference between the five colors of the three materials indicates interaction. The graph shows that Black, Brown, Light Brown, and Yellow had the same data pattern of ET < ESS < OMI, but the control did not have a distinct trend. The interaction effect refers to the difference in ET, ESS, and OMI between the five colors (including the control).

Visual analysis was conducted with 25 licensed dentists and 25 volunteers, each evaluating three trays containing 15 different types of randomly arranged samples. Each subject made 45 individual judgments. Table 2 and Table 3 present the data in a tabular format, detailing the percentages of “noticeable” and “non-noticeable” judgments. Among the materials analyzed, Black exhibited the highest proportion of “noticeable” judgments, with Brown, Light Brown, and Yellow following in a descending sequence. This was the same trend for both dentists and volunteers. Results for dentists showed that the ESS group was 99% Black, 79% Brown, and 59% Light Brown, revealing a difference of over 20% when compared to the OMI group’s Brown and Light Brown categories. The ET group’s Black percentage was 72%, indicating a divergence from the figures reported by OMI and ESS. ET was 72% for Black, a difference of more than 20% between OMI and ESS. Conversely, the differences between the materials were less than 10% for Yellow, 3% for ET, 1% for OMI, and 8% for ESS. In addition, 8% of the Control samples were judged “noticeable” by ET, 4% by OMI, and 3% by ESS.

Three plates were prepared for visual analysis, each with one sample from each of the 15 groups (3 materials × 5 colors). The judges comprised 25 licensed dentists and 25 volunteers. Out of these three plates, they chose the one with the most noticeable color difference. A total of 75 votes, with 3 votes each for each of the 25 participants. The voting results are presented in Table 4 and Table 5. OMI–Black received the most votes among dentists (58/75), followed by ESS–Black (12/75), OMI–Light Brown (3/75), OMI–Brown (1/75), and ET–Black (1/75). OMI–Black received the most votes among volunteers (42/75), followed by ESS–Black (28/75), OMI–Brown (2/75), ET–Black (2/75) and OMI–Light Brown (1/75).

## 4. Discussion

Resin composites are widely used in anterior and molar tooth because of dentin adhesive [27,28,29,30,31]. However, in clinical situations, recurrent caries may occur but not break away, delaying the detection of caries in some cases. Patients identify recurrent caries through color change, symptoms, and fissures, potentially postponing treatment.

Various composite resin products have been developed for different applications [1,2,3]. Recently, UC has garnered recognition as a single-shade resin that offers excellent color compatibility with teeth of diverse shades [15,16]. The first UC product, Omnichroma, was released in 2020, followed by Essensia Universal, ClearFill Majesty ES Flow Universal (Kuraray Noritake), and Beautiful Unishade (Shofu) [15,16,17,20,32,33]. Because UC is influenced by the color of the surrounding area, patients who can glance in the mirror and see changes in their teeth when recurring caries arise should anticipate seeing their dentist for early detection and treatment. Since UC is affected by the surrounding color, if patients can look at their teeth in a mirror and notice changes when recurrent caries occurs, it is expected to trigger a visit to the dentist, leading to early detection and early treatment.

In this study, several types of magic pens were prepared to simulate caries and composite resin was used instead of teeth. Usually, natural teeth are used in these experiments. However, the use of natural teeth is likely to result in large variations in dentin color, making evaluation between materials difficult. Therefore, we used a method of determining the visibility concerning the color of caries under conditions of uniform color using a magic pen and resin composite.

The color of caries differs between acute and chronic caries, with the softened dentin of acute caries being light yellow, whereas the softened dentin of chronic caries is brown to blackish brown [14]. Therefore, four different colors were used in this study.

Common spectrophotometers possess a minimum measurement range of 3 to 5 mm in diameter, which complicates the prediction of narrow regions. In this study, we used the image correction marker Casmatch (5 × 5 pixels range) for measurements.

CIEDE 2000 color difference formula was published in 2001 and is frequently used in dentistry to evaluate color differences, which was also used in this study [24,34,35,36,37,38].

The results of the photographic analysis showed significant differences between materials among almost all colors in the UC, but no significant differences among groups, including the Control, in EST of conventional resin composite. In addition, comparisons between colors showed identical data trends such as EST < ESS < OMI for Black, Brown, Light Brown, and Yellow, but no such clear trend was observed for the controls. This is the meaning of the interaction. Therefore, the null hypotheses (1) there is no color difference due to the color given and (2) there is no color difference due to material differences are rejected. Therefore, it can be said that the conventional resin composite did not affect the visualization of the cavity floor and the cavity floor may be visible in UC restorations. This may be because conventional resin composite achieves a good color match with the dentin by adding pigments [17,19,20], whereas UC blends naturally with tooth by reflecting or diffusing the surrounding color due to properties such as structural color [17] and light scattering [18].

For each color, OMI showed significant differences in all groups, while ESS showed significant differences in all groups except between Brown and Black, indicating different trends in results among the UCs.

Omnichroma is a UC based on structural color that develops by selectively interacting with the nanostructure of the tooth [17]. The uniform spacing and arrangement of spherical OMI particles facilitates the transmission of light throughout the restoration [21]. It reflects the color of the cavity surrounding the restoration and obtains a color match through blending [22].

Essentia Universal is a microhybrid UC with a blending effect [23]. The light transmission and scattering properties of ESS with microhybrid fillers are known to blend naturally with the surrounding tooth structure [24].

A previous study comparing the light transmittance characteristics of OMI and ESS found that ESS has a wider light transmission intensity distribution and better diffusion behavior than OMI [23]. Composite resins such as ESS have light diffusion properties that obscure the boundary with the surrounding tooth [39], whereas OMI has light diffusion and low opacity [22]. It is assumed that these differences in optical properties influenced the differences in results between the UCs.

The visual analysis included 25 licensed dentists and 25 volunteers who were not licensed dentists, dental hygienists, or dental technicians, with bilateral naked eye visual acuity of 0.6 or better or corrected visual acuity of 0.8 or better, and without color blindness or strong color weakness.

As in the photographic analysis, there is a high proportion of Black and Brown and a low proportion of Light Brown and Yellow. Especially in the case of Yellow, where almost all materials are in the same single-digit range, the color can be difficult to notice. Trends in the photographic analyses were similar to those in the visual analyses. Therefore, the null hypothesis of (3) the difference in the material affects the detection rate due to the difference in the operator was rejected. Regardless of the operator, UC tended to judge color differences as significant, at least for dark colors such as Black and Brown. This was the same trend for both dentists and volunteers.

Based on the above, UC restorations may be useful in detecting recurrent caries that may occur in the future. An individualized approach is recommended for adult caries, including secondary caries [5]. However, it is important for patients to be aware of recurrent caries and to visit a dentist, regardless of whether they choose to follow-up or to have restorations.

## 5. Conclusions

Within the limits of this study, the following can be concluded: Universal shade resin composite tends to reflect the color of the cavity more effectively than ordinary composite resins. Moreover, this is particularly applicable to structural color types compared to light-scattering types. Using universal shade resin composites makes it easier to detect color changes in cavities, perhaps leading to early identification of recurring caries with color changes.

## Figures and Tables

**Table 1:** Materials used in this study.

Material (Code)	Filler Type (Percent per Weight)	Composition	Percent per Weight	Manufacturer	Lot
Estelite Sigma Quick A3 (ET)	Supra-nano spherical filler (82)	2-Propenoic Acid, 2-Methyl-, (1- Methylethylidene) Bis [4,1- Phenyleneoxy (2-Hydroxy-3,1-Propanediyl)] Ester	10–30	Tokuyama Dental, Tokyo, Japan	J3051
		Triethylene Glycol Dimethacrylate	5–10		
		2,6-Di-tert-Butyl-p-Cresol	<1		
		p-Methoxyphenol	<1		
		Titanium Dioxide	<0.1		
Omnichroma (OMI)	Supra-nano spherical filler (79)	7,7,9(or7,9,9)-Trimethyl-4,13-Dioxo-3,14-Dioxa-5,12-Diazahexadecane-1,16-Diyl Bismethacrylate	10–30	Tokuyama Dental	019E89
		2,2′-Ethylenedioxydiethyl Dimethacrylate	1–5		
		2,6-Di-tert-Butyl-p-Cresol	<1		
		Dl-Bornane-2,3-Dione	<1		
		Mequinol	<1		
Essentia Universal (ESS)	Microhybrid filler (81)	Urethane Dimethacrylate	10–25	GC, Tokyo, Japan	2003091
		Ytterbium Trifluoride	5–10		
		(Octahydro-4,7-Methano-1H-Indenediyl) Bis (methylene) Bismethacrylate	2.5–5		
		2-(2H-benzotriazol-2-yl)-p-cresol	0.1–0.2		

**Table 2:** Percentage of “noticeable” calculated from visual analysis results ^1^.

		Black	Brown	Light Brown	Yellow	Control
	ET	72	47	23	3	8
Dentist	OMI	99	97	93	1	4
	ESS	99	79	59	8	3
	ET	72	29	27	4	15
Volunteer	OMI	100	77	60	5	12
	ESS	96	49	36	11	11

^1^ Values in the table are in percent (%).

**Table 3:** Percentage of “not noticeable” calculated from visual analysis for dentists results ^1^.

		Black	Brown	Light Brown	Yellow	Control
	ET	28	53	77	97	92
Dentist	OMI	1	3	7	99	96
	ESS	1	21	41	92	97
	ET	28	71	73	96	85
Volunteer	OMI	0	23	40	95	88
	ESS	4	51	64	89	89

^1^ Values in the table are in percent (%).

**Table 4:** Voting results of the visual analysis for dentists.

Rank	Material	Color	Votes
1st	OMI	Black	58
2nd	ESS	Black	12
3rd	OMI	Light Brown	3
4th	OMI	Brown	1
5th	ET	Black	1

**Table 5:** Voting results of visual analysis for volunteers.

Rank	Material	Color	Votes
1st	OMI	Black	42
2nd	ESS	Black	28
3rd	OMI	Brown	2
4th	ET	Black	2
5th	OMI	Light Brown	1

## Data Availability

The data presented in this study are available on request from the corresponding author due to privacy.

## Appendix A

A total of 25 licensed dentists who were at least 24 years old, less than 40 years post-dental school, with bilateral naked eye visual acuity ≥ 0.6 or corrected visual acuity ≥ 0.8, and without color blindness or strong color weakness were included in the study. The distributions of participants’ gender, age, and clinical experience are shown in Table A1, Table A2 and Table A3.

**Table A1:** Gender Distribution of the Participants (Dentists).

	Male	Female
Number	13	12

**Table A2:** Clinical Experience Distribution of the Participants (Dentists).

	Resident (<1 Y)	Grad Student (1–5 Y)	Junior Faculty ^1^	Senior Faculty ^2^
Number	7	8	4	6

^1^ Instructor class less than 10 years after graduation. ^2^ Instructor class more than 10 years after graduation.

**Table A3:** Age Distribution of the Participants (Dentists).

	20 s	30 s	40 s	50 s
Number	13	6	2	4

A total of 25 volunteers who were at least 20 years old, with bilateral naked eye visual acuity ≥ 0.6 or corrected visual acuity ≥ 0.8, and without color blindness or strong color weakness, and were not licensed as dentists, dental hygienists, or dental technicians were included in the study. The distributions of participants’ gender and age are shown in Table A4 and Table A5.

**Table A4:** Gender Distribution of the Participants (Volunteers).

	Male	Female
Number	17	8

**Table A5:** Age Distribution of the Participants (Volunteers).

	20 s	30 s	40 s	50 s	60 s	70 s
Number	14	4	2	2	2	1
